# Cognitive dedifferentiation as a function of cognitive impairment in the ADNI and MemClin cohorts

**DOI:** 10.18632/aging.203108

**Published:** 2021-05-26

**Authors:** John Wallert, Anna Rennie, Daniel Ferreira, J-Sebastian Muehlboeck, Lars-Olof Wahlund, Eric Westman, Urban Ekman, and ADNI consortium, and MemClin steering committee

**Affiliations:** 1 Center for Alzheimer Research, Division of Clinical Geriatrics, Department of Neurobiology, Care Sciences, and Society, Karolinska Institutet, Stockholm, Sweden; 2 Centre for Psychiatry Research, Department of Clinical Neuroscience, Karolinska Institutet, Stockholm, Sweden; 3 Theme Aging, Karolinska University Hospital, Stockholm, Sweden; 4 Department of Neuroimaging, Centre for Neuroimaging Sciences, Institute of Psychiatry, Psychology and Neuroscience, King´s College, London, UK; 5 Medical Unit Medical Psychology, Allied Health Professionals Function, Karolinska University Hospital, Stockholm, Sweden

**Keywords:** aging, cognitive decline, dotage, neurodegeneration, prodromal dementia

## Abstract

The cause of cognitive dedifferentiation has been suggested as specific to late-life abnormal cognitive decline rather than a general feature of aging. This hypothesis was tested in two large cohorts with different characteristics. Individuals (n = 2710) were identified in the Alzheimer’s Disease Neuroimaging Initiative (ADNI) research database (n = 1282) in North America, and in the naturalistic multi-site MemClin Project database (n = 1223), the latter recruiting from 9 out of 10 memory clinics in the greater Stockholm catchment area in Sweden. Comprehensive neuropsychological testing informed diagnosis of dementia, mild cognitive impairment (MCI), or subjective cognitive impairment (SCI). Diagnosis was further collapsed into cognitive impairment (CI: MCI or dementia) vs no cognitive impairment (NCI). After matching, loadings on the first principal component were higher in the CI vs NCI group in both ADNI (53.1% versus 38.3%) and MemClin (33.3% vs 30.8%). Correlations of all paired combinations of individual tests by diagnostic group were also stronger in the CI group in both ADNI (mean inter-test r = 0.51 vs r = 0.33, p < 0.001) and MemClin (r = 0.31 vs r = 0.27, p = 0.042). Dedifferentiation was explained by cognitive impairment when controlling for age, sex, and education. This finding replicated across two separate, large cohorts of older individuals. Knowledge that the structure of human cognition becomes less diversified and more dependent on general intelligence as a function of cognitive impairment should inform clinical assessment and care for these patients as their neurodegeneration progresses.

## INTRODUCTION

**Figure 1:**
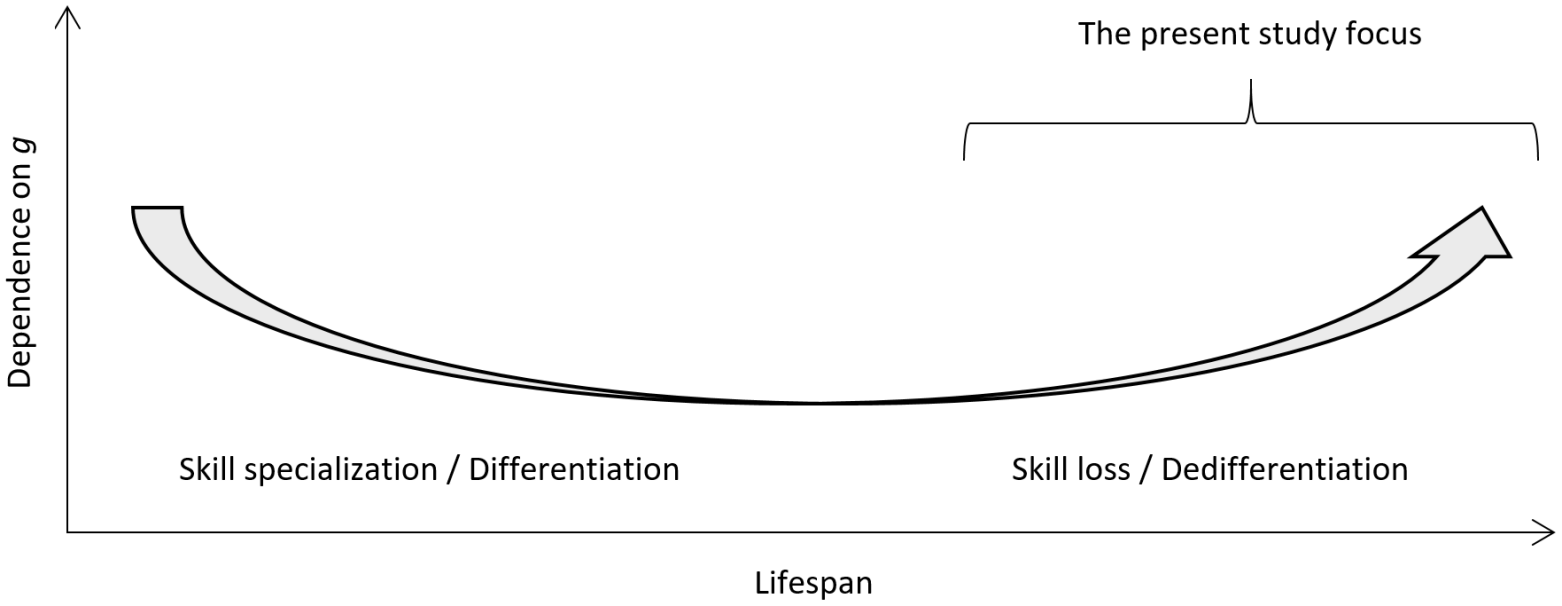
**Conceptual plot for the degree of dependence of cognitive test scores on general intelligence (*g*) as a function of cognitive development and specialization in young age, stability in adult age, and decline in old age.** The present study focus is highlighted.

The age *dedifferentiation* hypothesis suggests that as humans age we become more reliant on general intelligence (*g*) for the different cognitive functions [1]. This is a somewhat contested idea in the geriatric field where it has been suggested that underlying pathology accounts for dedifferentiation [2]. Based on early research on dedifferentiation of cognitive abilities in later life, [3] this hypothesis suggests that the influence of *g* on cognitive test performance increases as the biological constraint that comes with old age renders specific cognitive abilities more similar [4]. When dedifferentiation is combined with the (i) known *differentiation* of cognitive functioning that occurs due to skill specialization from young age to adulthood, and (ii) stability of adult cognitive functioning from 18-65 years of age, [5] a conceptual U-type lifespan plot can be drawn (Figure 1) similar to Craik and Bialystok [6].

In the neuroscience literature, age-related neural dedifferentiation is a fairly robust phenomenon [7]. However, ascribing aging itself causal agency for dedifferentiation is probably too coarse [6]. Studies have shown substantial cognitive dedifferentiation in older samples but only in those with suspected [8] or diagnosed [9] abnormal neurodegenerative decline. An age *indifferentiation* hypothesis has also been proposed that stresses the stability of cognitive ability over time [10]. Aside from aging, other potential causes of dedifferentiation have been described, such as educational attainment [11]. Control for education and other potential confounders is needed if one wants to estimate the unique contribution of neurodegeneration to dedifferentiation [8].

Dedifferentiation seems in part specific to abnormal cognitive decline and, consequently, of specific interest for clinical geriatric populations. Among patients with subjective cognitive complaints seeking healthcare, subjective cognitive impairment (SCI) is distinguished from cognitive impairment (CI) – i.e. either Mild Cognitive Impairment (MCI) or dementia diagnosis – through comprehensive multidisciplinary investigation at a specialized hospital unit, usually a Memory Clinic [12] To our knowledge, the dedifferentiation hypothesis has not been tested in a large, ecologically valid and representative sample of memory clinic patients. Dedifferentiation in these patients, whom have all undergone the memory clinic investigation and been diagnosed as either CI (MCI or dementia) or No CI (SCI), can be studied and the association of CI with dedifferentiation estimated.

The present study thus tested the dedifferentiation hypothesis as a function of CI in two cohorts, each of considerable size and each including both individuals with CI and NCI, but with important differences regarding their geographical location (ADNI: North America [13], MemClin: Sweden) [12], diagnostic setting (research setting; clinical setting), sample selection, and data characteristics. After controlling for age, education, and sex, we hypothesized that as CI comes into play, dedifferentiation is greater and the influence of *g* on cognitive test performance is higher. We also expected dedifferentiation to be more clear in the ADNI database compared to MemClin since ADNI applied a set of additional exclusion criteria to a priori differentiate diagnostic groups whereas MemClin did not have such exclusion but instead by design includes patients that are the most difficult to diagnostically differentiate requiring full memory clinic investigation.

## RESULTS

Summary statistics for patients subgrouped by dataset and by group are available in Table 1. Within each dataset, patients with CI were older, had completed fewer years of education, more likely male, and performed worse on psychometric tests, compared to NCI patients. Across datasets, patients were similar in age but ADNI patients had more years of education. ADNI patient counts were more evenly dispersed across groups relative to MemClin. In addition, differences in psychometric performance across CI and NCI groups were slightly more pronounced in ADNI compared to MemClin.

**Table 1:** Descriptive statistics in ADNI and MemClin within each dataset stratified as CI and NCI.

	**ADNI**			**MemClin**	
	**CI (n = 884)**	**NCI (n = 398)**		**CI (n = 1071)**	**NCI (n = 152)**
Age (yrs)	74.33 (7.40)	74.24 (5.68)		78.32 (6.00)	75.32 (5.70)
Education (yrs)	15.76 (2.86)	16.40 (2.68)		12.22 (3.59)	13.54 (3.74)
Male sex	517 (58.8)	188 (47.2)		551 (51.4)	61 (40.1)
Diagnosis, three class					
Dementia*/AD	503 (56.9)	0 (0.0)		331 (30.9)	0 (0.0)
MCI	381 (43.1)	0 (0.0)		740 (69.1)	0 (0.0)
CN/SCI	0 (0.0)	398 (100.0)		0 (0.0)	152 (100.0)
Key psychometric tests					
AVLT 1	4.05 (1.57)	5.48 (1.73)		3.55 (1.68)	5.64 (1.73)
AVLT delayed recall	2.64 (3.42)	7.85 (3.76)		3.98 (3.22)	10.11 (2.96)
TMT B / TMT 3	142.97 (81.77)	82.86 (41.07)		84.87 (38.93)	46.62 (16.62)
MMSE	26.30 (2.73)	29.07 (1.16)		26.67 (2.46)	28.91 (1.36)

Data are mean (SD) and count (%). Psychometric tests are raw scores. *MemClin includes other subtypes, most frequently AD, vascular dementia, and mixed dementia. ADNI, Alzheimer’s Disease Neuroimaging Initiative; MemClin, Memory Clinic project; AD, Alzheimer’s disease; AVLT, Rey Auditory Verbal Learning Test; CI, Cognitive impairment; NCI, No cognitive impairment; CN, Cognitively normal; MCI, Mild cognitive impairment; MMSE, Mini Mental State Examination; SCI, Subjective cognitive impairment; TMT, Trail-Making Test.

Table 2 shows a higher proportion of variance in psychometric test scores explained by PC1 for the CI group vs NCI, across both ADNI and MemClin. Applying the Kaiser rule retained one less PC for the CI group vs NCI in ADNI. However, equal amount of PCs were retained across groups in MemClin. Regarding individual PC1 test loadings for key cognitive domains, the averages were slightly higher and less variable across domains for the CI group vs NCI in ADNI but not in MemClin.

**Table 2:** Principal component analysis (PCA) in the separate ADNI and MemClin datasets by CI vs NCI groups after matching.

	**ADNI**			**MemClin**	
	**CI (n = 392)**	**NCI (n = 392)**		**CI (n = 143)**	**NCI (n = 143)**
% variance explained by PC1	53.1	38.3		33.3	30.8
N factors with eigenvalues > 1	2	3		5	5
Individual test loadings on PC1					
Working memory (AVLT 1)	0.24	0.27		0.28	0.27
Episodic memory (AVLT delayed recall; AVLT 5)	0.26	0.32		0.28	0.29
Executive function (TMT B; TMT 3)	0.24	0.13		0.18	0.19
General (MMSE)	0.25	0.10		0.20	0.18

Patients have been propensity score matched 1:1 without replacement on age, education and sex. PCA was thereafter performed through single value decomposition. Values are calculated with imputed and propensity score matched data controlling for age, education, and sex. ADNI, Alzheimer’s Disease Neuroimaging Initiative; MemClin, Memory Clinic Project; AVLT, Rey Auditory Verbal Learning Test; CI, Cognitive impairment; NCI, No cognitive impairment; MMSE, Mini Mental State Examination.

**Figure 2:**
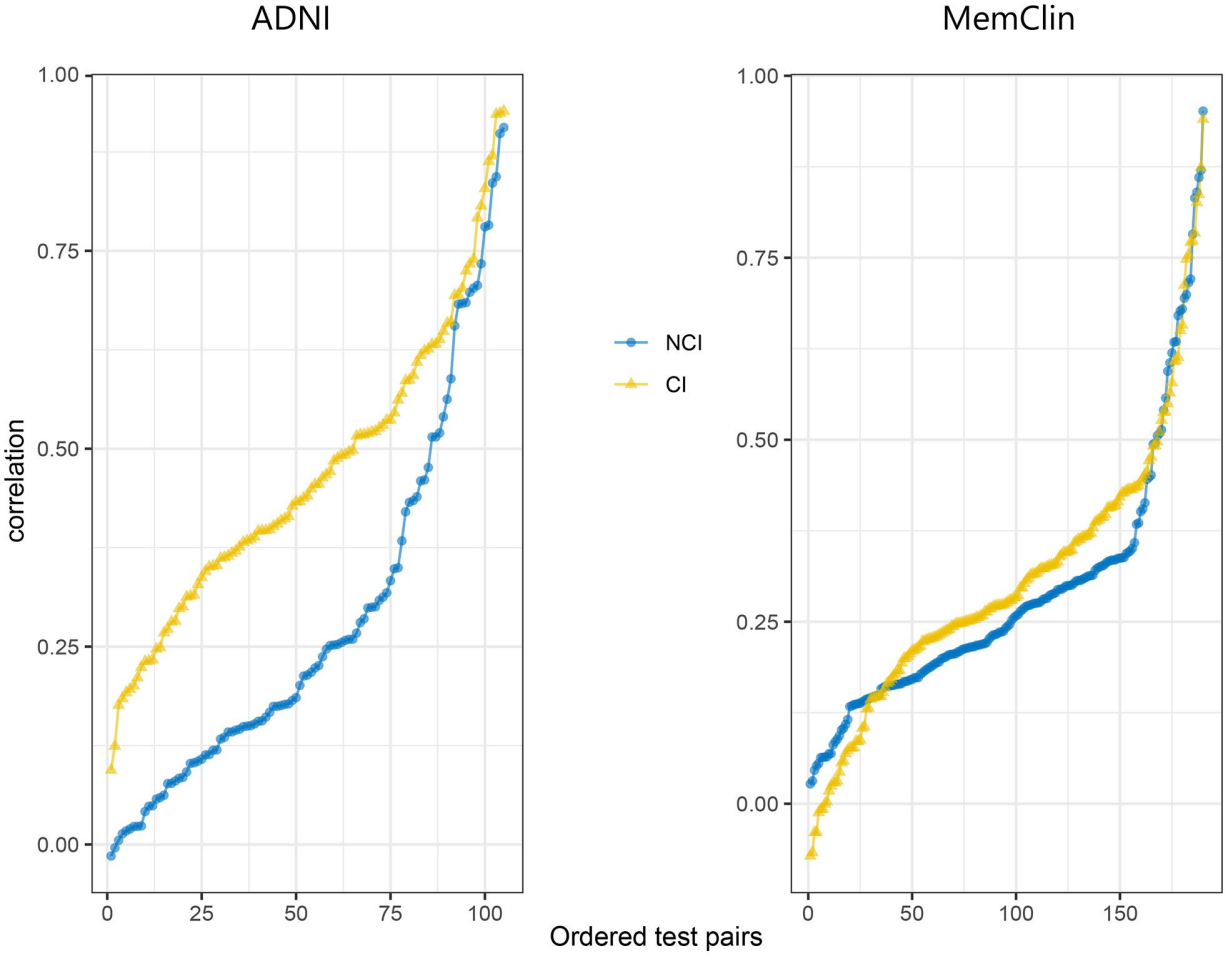
**Sorted correlation strengths across all informative test pairs in the ADNI (n = 105) and MemClin (n = 190) datasets.** Notice that inverse test scores (e.g. TMT) had been rescaled prior and that the assumption of positive manifold is slightly violated (a few negative correlations), possibly due to stochasticity. ADNI, Alzheimer’s Disease Neuroimaging Initiative; MemClin, Memory Clinic Project; CI, Cognitive Impairmen; NCI, No Cognitive Impairment; TMT, Trail-Making Test.

All informative test pair correlations (105 for ADNI; 190 for MemClin) sorted from weakest to strongest magnitude stratified by database and group are depicted in Figure 2 showing dedifferentiation by impairment in both datasets, i.e. generally stronger linear associations between test pairs among CI vs NCI patients (ADNI: mean inter-test r = 0.51 vs r = 0.33, p < 0.001) and MemClin: r = 0.31 vs r = 0.27, p = 0.042). We also see that the dedifferentiation pattern is more pronounced in ADNI compared to MemClin but present in both.

## DISCUSSION

After controlling for age, education and sex through propensity score matching, dedifferentiation was specifically associated with cognitive impairment in the present study. This was shown in two large, high-quality cohorts that differed in several aspects; the research oriented ADNI and the naturalistic cohort MemClin. Our findings are in line with previous studies showing age-related dedifferentiation of cognitive abilities, yet does not focus on aging with respect to dedifferentiation. Instead, our findings corroborate previous research highlighting the role of impairment of cognitive functions as explanatory for cognitive dedifferentiation in the latter portion of life, rather than explained by aging per se [8, 9].

Research on dedifferentiation, as the phenomenon is defined and investigated with data, predominantly focuses on cognitive change processes that are global in their nature. There are however more specific and also subtle changes to human cognition in late life. Cognitive reorganization has been suggested, [14] as well as findings suggesting that differentiation and dedifferentiation processes can be simultaneously ongoing as part of a cognitive restructuring process that is compensatory to newly developed cognitive deficiency [15]. There is also the concept of cognitive reserve suggesting interindividual differences with regards to the amount of complex cognitive activity experienced during life. This experience in turn determines interindividual differences in accumulated cognitive reserve which functions as a flexible and active buffer to cognitive decline, for some but not for others, as neurodegeneration progresses [16]. There are likely both fixed and modifiable factors producing variability in cognitive reserve across individuals that may in turn influence dedifferentiation caused by neurodegeneration. In our study we did control for education and thus also controlled for cognitive reserve by proxy so the present findings can hardly be explained by cognitive reserve. One can conclude that more research on these largely interlinked cognitive processes and their relationship with dedifferentiation is needed.

### Clinical perspective

To our knowledge, this is the first time cognitive impairment as explanatory variable for dedifferentiation is found in two large and separate cohorts in which each patient’s cognition has been thoroughly examined and diagnosed. The present study therefore puts particular emphasis on the dedifferentiation phenomenon in the context of geriatric care and its patient population in abnormal cognitive decline, i.e. patients with MCI or dementia.

One important issue that the present study highlights pertains to the cognitive profile of a patient, and whether this profile of performance on psychometric tests is relatively similar across cognitive domains gauged by the tests. Clinicians often reason that an “even” profile is a sign of healthy cognitive functioning, and vice versa. The present study problematizes such reasoning as it suggests that an even cognitive profile can de facto be due to dedifferentiation of cognitive abilities. Most clinicians are well-aware that a cognitive test profile that is similar across domains but significantly worse across these domains relative to normative test scores usually signifies some form of cognitive decline. If however dedifferentiation presents itself early on for a patient, it might obscure a serious condition if the clinician applies the “even profile” heuristic when presented with a cognitive profile that is a bit lower in performance relative to applicable test norms but similar across cognitive domains. Simply because AD is very common, and because the classic AD profile is uneven with fairly distinct underperformance on episodic memory tests, does not allow for diagnostic heuristics to be applied insofar that possible dedifferentiation is ignored. Further, if a cognitive profile of a patient is even because of dedifferentiation and then also coupled with inadequately estimated premorbid cognition, proper diagnosis of MCI could be substantially delayed for patients initially classified as cognitively healthy or with only subjective cognitive impairment.

Cognitive status conditions are difficult to diagnose and require thorough examination by a multidisciplinary team at a memory clinic to achieve sufficient diagnostic accuracy for the most difficult to separate patients. In clinical practice, stronger dedifferentiation with progressing neurodegeneration means that impaired patients depend to a greater extent on their general intelligence because their task specific skills developed earlier in life are deteriorating. This suggests that important lessons are to be learned regarding how these patients become, for instance, cognitively overburdened in concrete daily situations, during which they previously could offload their cognitive ability through skill heuristics but which are no longer accessible, or not as easily accessible to them.

### Limitations and strengths

All observational research is limited by possibly remaining residual confounding. This includes our study, since there may be other factors than cognitive impairment that produces dedifferentiation of cognitive abilities in late life. Factors such as biological age [15], cognitive reserve [17], or brain compensation [18] might also produce dedifferentiation, and we cannot exclude the possibility that compensatory mechanisms co-exist with cognitive impairment, especially in the early phases. We were not able to directly control for all these factors in the present study, but through propensity score matching on age, education, and sex, it is reasonable to assume that we control for the bulk of such possible confounding indirectly. Control for premorbid ability was not deemed necessary as years of education was controlled for and is considered a good proxy for premorbid ability. Moreover, the different prevailing retrospective methods for estimating premorbid ability; using education and demographics, or semantic knowledge test performance (e.g. WAIS-IV Information), or a specific pronunciation type test (e.g. NART [19], ISW [20]), have their respective limitations such as overestimation and conceptual biases. More research is likely needed in which other potential confounders are controlled for, simultaneously being wary of the modelling pitfall of overadjustment bias, i.e. controlling for intermediary variables situated on the suggested causal path from cognitive impairment to dedifferentiation. Our study was also limited by data being cross-sectional. There are longitudinal measurements on some variables in both ADNI and MemClin but a more complete recording of psychometric performance across time is needed to study dedifferentiation over time within individuals. Another important feature in our study was the multiple dimensions for which the ADNI and MemClin cohorts differ. For instance, ADNI is a North American research database, employing in part different psychometric tests, brain imaging techniques, diagnostic methods, and patient inclusion procedures compared to the Swedish MemClin database. Because of these differences, the two datasets could not be combined and analysed as a whole. We could however use the strengths of this study feature by investigating, and also finding, dedifferentiation in the two datasets separately, leveraging their individually fairly large size and generally high quality of measurements. Another important difference relates to differing sampling procedures for which ADNI deliberately sampled healthy controls whereas MemClin controls where SCI patients. The pattern of dedifferentiation was also more pronounced in ADNI compared to MemClin. Importantly, MemClin is a new large-scale naturalistic database with high ecological validity and generalizability to the clinical geriatric population in its specialized care setting provides both a rare strength, and a complement to the excellent ADNI database for conducting such studies as the present one.

## CONCLUSIONS

Dedifferentiation of cognitive abilities in late life was investigated and identified in two large and independent cohorts. Through adjustment for age, education, and sex, an independent association of cognitive impairment on dedifferentiation was found. The meta-cognitive aspect of dedifferentiation is important and should be accounted for by clinicians as they diagnose, treat, and care for their patients.

## MATERIALS AND METHODS

### Study population

We included patients from the Alzheimer’s Disease Neuroimaging Initiative (ADNI, n = 1282) [13] and the Memory Clinic Project (MemClin, n = 1223) [12]. The diagnostic procedures for ADNI [21] and MemClin have previously been described [12]. For ADNI, the diagnosis procedure relied on subjective and objective cognitive measures but was independent of biomarker information. For MemClin, diagnosis was determined at multidisciplinary consensus meetings based on cognitive testing, neurological examination, brain imaging, biomarkers and other routine clinical measures as available [12, 22, 23]. The diagnosed cognitive statuses of dementia, MCI, and SCI were collapsed into two classes: CI and NCI. CI represents impairment of clinically diagnosed severity whereas NCI represents no objective impairment.

### Cognitive variables

The administered neuropsychological batteries of ADNI and MemClin are described elsewhere [12, 14] and covered similar cognitive domains and subdomains with some differences by original design. Importantly however, across main analysis comparisons of dedifferentiation in on CI vs NCI variables were identical in each database. Unless further defined, psychometric tests are total raw scores. Some psychometric variables were reverse coded as needed. Psychometric tests are separated with semi-colon (;) and subtests belonging to the same test are separated with dash (/) as follows.

From ADNI we selected patient performance on 15 variables: the Mini Mental State Examination (MMSE); Clock test; Copy test; Rey Auditory Verbal Learning Test (AVLT) trial 1/2/3/4/5/total score/delayed recall/recognition; semantic fluency (animals); Trail-Making Test (TMT) A/B; and Boston Naming Test spontaneous recall.

From MemClin we selected 20 variables: MMSE; Wechsler Adult intelligence Scale 4^th^ edition (WAIS-IV) Information; Block design; Digit Span total/forward/backward; AVLT 1/2/3/4/5/total score; Rey Complex Figure copy; Delis-Kaplan Executive Function System (D-KEFS) Verbal fluency FAS/semantic fluency/shifting (correct answers)/shifting (correct shiftings); and TMT 1/2/3. TheHiveDB was used for MemClin data management [24].

### Statistical analyses

All analyses were mirrored and run separately in both ADNI and MemClin. Due to missing data some variables were excluded (cut-off > 25% missing). Remaining missing data sorted by % missing of total (in ADNI: TMT B [1.9], NART [0.7], sex [0.3], age [0.3], edu [0.3] and in MemClin: AVLT total [23.8], D-KEFS TMT 3 [22.8], RCFT copy [22.6], WAIS-IV Block design [20.9], D-KEFS TMT 1 [20.7] was deemed acceptable, assumed to be missing at random (MAR) and imputed with *k* Nearest Neighbour (kNN) [25, 26] applying the unweighted Gower distance metric [27] with *k* set to three.

**Figure 3:**
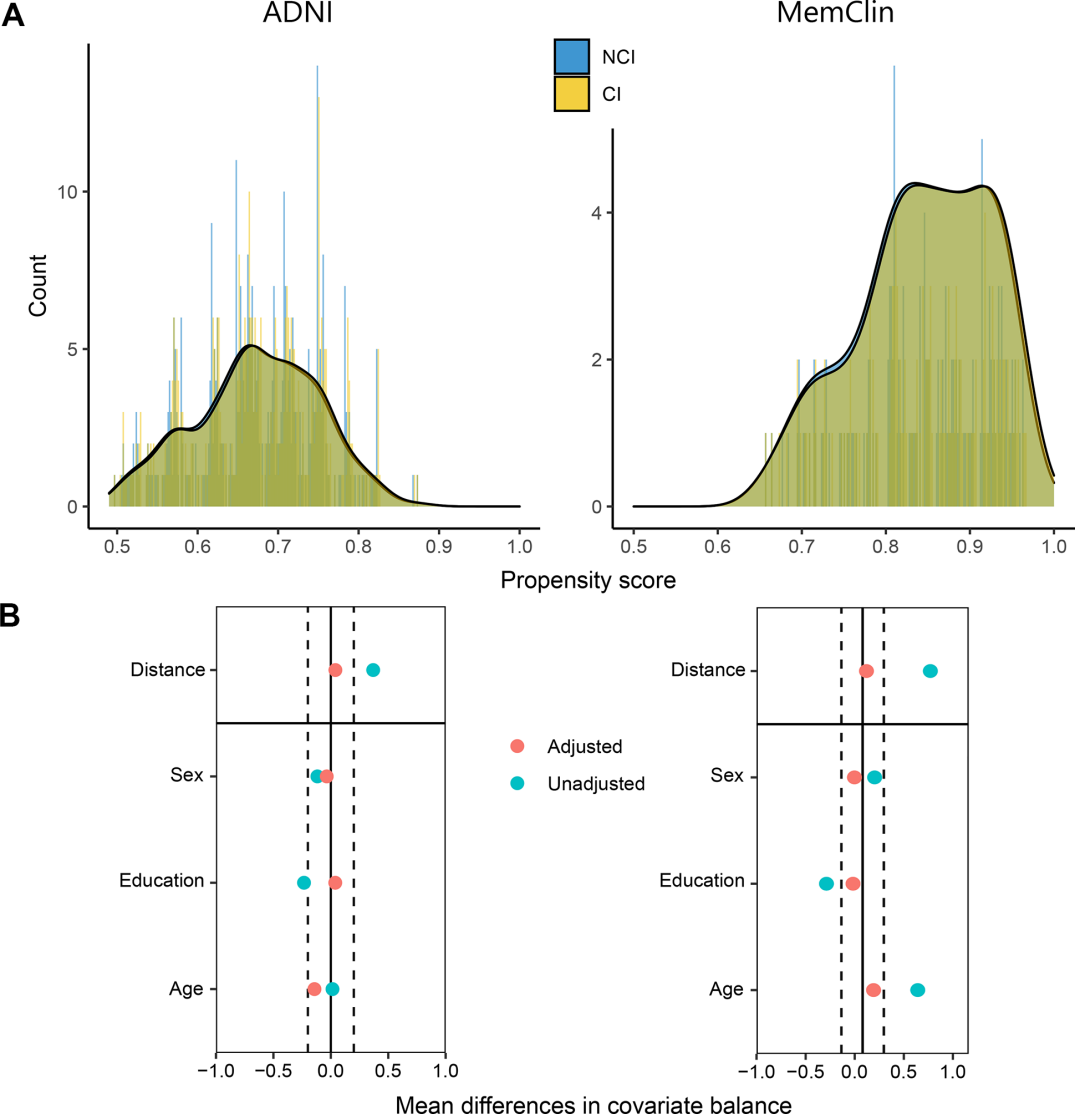
**Propensity score matching diagnostics in the ADNI and MemClin datasets.** (**A**) Patient counts after matching are shown as a function of their individual propensity score and overlayed with density plots, stratified by level of cognitive impairment. (**B**) Mean distance followed by single-covariate balance by group calculated before (Unadjusted) and after (Adjusted) matching. ADNI matched: n CI= 392, n NCI = 392. MemClin matched: n CI = 143, n NCI = 143. ADNI, Alzheimer’s Disease Neuroimaging Initiative; MemClin, Memory Clinic Project; CI, cognitive impairment; NCI, no cognitive impairment.

To control for confounding of the Dedifferentiation ~ CI association, propensity score matching [28, 29] was performed prior to main analysis. A logit model was constructed estimating the probability for CI with age at examination, years of education, and sex as predictors. Exposed (CI) where thereafter propensity score matched 1:1 with unexposed (NCI) without replacement on this probability applying SD = 0.05 caliper width. Figure 3 plots the matching diagnostics and resulting across-group balance in the confounders.

### Main analysis

After matching, principal component analysis (PCA) was performed on the mean centered and unit variance scaled psychometric variables through single value decomposition. Percentage explained variance in psychometric variables by the first unrotated component (PC1), a proxy for general cognitive ability, was compared across the matched CI and NCI groups expecting a smaller % explained variance by PC1 among scores in the CI vs the NCI group. We also report n components with eigenvalues > 1 (Kaiser criterion) expecting fewer selected components by this criterion for CI vs NCI. Loadings on PC1 by key psychometric tests that tap central cognitive domains were expected to be higher in CI vs NCI groups. Supplementary Tables 1, 2 show PCA analysis by subgroups after propensity score matching for both the ADNI and the MemClin cohort.

Correlation coefficients calculated across all informative pairings of psychometric variables were thereafter sorted from weakest to strongest and assessed by t-test and also by visual inspection comparing the strengths of correlations across matched CI and NCI groups expecting average test-pair correlations to be higher in the CI vs the NCI group.

### Additional statistics

Unless further explained, bivariate summary statistics are presented as mean (SD) for numeric variables and count (%) for factors. Statistical significance was set to 5%.

Analyses were performed in R version 4.0.1 using packages caret, cobalt, corrplot, data.table, dummies, factoextra, FactoMineR, foreign, ggplot2, gridExtra, haven, MatchIt, matrixStats, mice, readxl, tableone, and VIM.

## Supplementary Material

Supplementary Tables

## References

[1] Reinert G. Comparative Factor Analytic Studies of Intelligence Throughout the Human Life-span. The West Virginia University Conference on Life-Span Developmental Psychology; Morgantown, West Virginia University 1970.

[2] Dodge HH, Marson DC. Illuminating cognitive dedifferentiation at the end of life. Neurology. 2012; 78:1110–11. 10.1212/WNL.0b013e31824f80b9 22491863

[3] Balinsky B. An analysis of the mental factors of various age groups from nine to sixty. Genetic Psychology Monographs. 1941; 23:191–234.

[4] Balsamo M, Romanelli R, Saggino A. The de-differentiation hypothesis in normal elderly persons. Percept Mot Skills. 2010; 110:85–88. 10.2466/PMS.110.1.85-88 20391873

[5] Rönnlund M, Sundström A, Nilsson LG. Interindividual differences in general cognitive ability from age 18 to age 65 years are extremely stable and strongly associated with working memory capacity. Intelligence. 2015; 53:59–64.

[6] Craik FI, Bialystok E. Cognition through the lifespan: mechanisms of change. Trends Cogn Sci. 2006; 10:131–38. 10.1016/j.tics.2006.01.007 16460992

[7] Koen JD, Rugg MD. Neural Dedifferentiation in the Aging Brain. Trends Cogn Sci. 2019; 23:547–59. 10.1016/j.tics.2019.04.012 31174975 PMC6635135

[8] Batterham PJ, Christensen H, Mackinnon AJ. Comparison of age and time-to-death in the dedifferentiation of late-life cognitive abilities. Psychol Aging. 2011; 26:844–51. 10.1037/a0023300 21534687

[9] Wilson RS, Segawa E, Hizel LP, Boyle PA, Bennett DA. Terminal dedifferentiation of cognitive abilities. Neurology. 2012; 78:1116–22. 10.1212/WNL.0b013e31824f7ff2 22491858 PMC3320052

[10] Juan-Espinosa M, García LF, Escorial S, Rebollo I, Colom R, Abad FJ. Age dedifferentiation hypothetis: Evidence from the WAIS III. Intelligence. 2002; 30:395–408.

[11] Christensen H. What cognitive changes can be expected with normal ageing? Aust N Z J Psychiatry. 2001; 35:768–75. 10.1046/j.1440-1614.2001.00966.x 11990887

[12] Ekman U, Ferreira D, Muehlboeck JS, Wallert J, Rennie A, Eriksdotter M, Wahlund LO, Westman E. The MemClin project: a prospective multi memory clinics study targeting early stages of cognitive impairment. BMC Geriatr. 2020; 20:93. 10.1186/s12877-020-1478-3 32138686 PMC7059672

[13] Mueller SG, Weiner MW, Thal LJ, Petersen RC, Jack C, Jagust W, Trojanowski JQ, Toga AW, Beckett L. The Alzheimer’s disease neuroimaging initiative. Neuroimaging Clin N Am. 2005; 15:869–77. 10.1016/j.nic.2005.09.008 16443497 PMC2376747

[14] Bock O, Haeger M, Voelcker-Rehage C. Structure of executive functions in young and in older persons. PLoS One. 2019; 14:e0216149. 10.1371/journal.pone.0216149 31071104 PMC6508866

[15] Gonzalez-Burgos L, Hernández-Cabrera JA, Westman E, Barroso J, Ferreira D. Cognitive compensatory mechanisms in normal aging: a study on verbal fluency and the contribution of other cognitive functions. Aging (Albany NY). 2019; 11:4090–106. 10.18632/aging.102040 31232698 PMC6628999

[16] Stern Y. Cognitive reserve. Neuropsychologia. 2009; 47:2015–28. 10.1016/j.neuropsychologia.2009.03.004 19467352 PMC2739591

[17] Gonzalez-Burgos L, Barroso J, Ferreira D. Cognitive reserve and network efficiency as compensatory mechanisms of the effect of aging on phonemic fluency. Aging (Albany NY). 2020; 12:23351–78. 10.18632/aging.202177 33203801 PMC7746387

[18] Gonzalez-Burgos L, Pereira JB, Mohanty R, Barroso J, Westman E, Ferreira D. Cortical Networks Underpinning Compensation of Verbal Fluency in Normal Aging. Cereb Cortex. 2021. [Epub ahead of print]. 10.1093/cercor/bhab052 33866353 PMC8258442

[19] Nelson HE. National Adult Reading Test (NART): For the Assessment of Premorbid Intelligence in Patients with Dementia: Test Manual, NFER-Nelson, Windsor, UK. 1982.

[20] Almkvist O, Tallberg IM. Cognitive decline from estimated premorbid status predicts neurodegeneration in Alzheimer’s disease. Neuropsychology. 2009; 23:117–24. 10.1037/a0014074 19210039

[21] Petersen RC, Aisen PS, Beckett LA, Donohue MC, Gamst AC, Harvey DJ, Jack CR Jr, Jagust WJ, Shaw LM, Toga AW, Trojanowski JQ, Weiner MW. Alzheimer’s Disease Neuroimaging Initiative (ADNI): clinical characterization. Neurology. 2010; 74:201–09. 10.1212/WNL.0b013e3181cb3e25 20042704 PMC2809036

[22] Wallert J, Westman E, Ulinder J, Annerstedt M, Terzis B, Ekman U. Differentiating Patients at the Memory Clinic With Simple Reaction Time Variables: A Predictive Modeling Approach Using Support Vector Machines and Bayesian Optimization. Front Aging Neurosci. 2018; 10:144. 10.3389/fnagi.2018.00144 29881341 PMC5972201

[23] Wallert J, Ekman U, Westman E, Madison G. The worst performance rule with elderly in abnormal cognitive decline. Intelligence. 2017; 64:9–17.

[24] Muehlboeck JS, Westman E, Simmons A. TheHiveDB image data management and analysis framework. Front Neuroinform. 2014; 7:49. 10.3389/fninf.2013.00049 24432000 PMC3880907

[25] Beretta L, Santaniello A. Nearest neighbor imputation algorithms: a critical evaluation. BMC Med Inform Decis Mak. 2016 (Suppl 3); 16:74. 10.1186/s12911-016-0318-z 27454392 PMC4959387

[26] Jönsson P, Wohlin C, editors. An Evaluation of k-Nearest Neighbour imputation Using Likert Data. 10th International Symposium on Software Metrics; 2004: IEEEXplore.

[27] Kowarik A, Templ M. Imputation with R package VIM. J Stat Softw. 2016; 74:1–16.

[28] Rosenbaum PR, Rubin DB. The Central Role of the Propensity Score in Observational Studies for Causal Effects. Biometrika. 1983; 70:41–55.

[29] Ho D, Kosuke I, King G, Stuart E. Matching as Nonparametric Preprocessing for Reducing Model Dependence in Parametric Causal Inference. Political Analysis. 2007; 15:199–236.

