# Supplementary material for: Cognitive dedifferentiation as a function of cognitive impairment in the ADNI and MemClin cohorts
Source: Aging (Albany NY). 2021 May 26;13(10):13430–42. doi: 10.18632/aging.203108 (PMC8202862; doi:10.18632/aging.203108)
Supplement: Supplementary Tables [file aging-13-203108-s001.pdf]

SUPPLEMENTARY TABLES

Supplementary Table 1. Principal component analysis by subgroups after propensity score matching, ADNI.

	PC1	PC2	PC3	PC4	PC5	PC6
**CI group (n = 392)**						
Standard deviation	2.8219	1.3263	0.9675	0.9160	0.8132	0.7417
Proportion of variance	0.5309	0.1173	0.0624	0.0559	0.0441	0.0367
Cumulative proportion of variance	0.5309	0.6481	0.7106	0.7665	0.8109	0.8473
Individual test loadings						
MMSE	-0.2520	0.1396	-0.1920	0.1648	0.0883	-0.3796
Clock test	-0.2030	0.3575	0.1946	0.2835	0.1469	-0.5835
Copy test	-0.1161	0.3926	0.5224	0.3829	-0.5205	0.2608
AVLT 1	-0.2438	-0.0350	0.2880	-0.5600	-0.1158	-0.1179
AVLT 2	-0.3024	-0.1348	0.1988	-0.2179	0.0112	0.0164
AVLT 3	-0.3122	-0.1978	0.1170	-0.0538	-0.0688	-0.0297
AVLT 4	-0.3178	-0.1944	0.0405	0.0427	-0.0366	-0.0579
AVLT 5	-0.3133	-0.2110	-0.007	0.0967	0.0227	-0.0274
AVLT total	-0.3376	-0.1873	0.1156	-0.0969	-0.0328	-0.0432
AVLT delayed	-0.2597	-0.3144	-0.0800	0.2842	-0.0341	0.1792
AVLT recognition	-0.2380	-0.1628	-0.2310	0.4567	0.1842	0.2374
Semantic fluency Animals	-0.2447	0.2077	-0.3321	-0.1575	-0.2641	0.3054
TMT A	-0.2110	0.3900	0.0924	-0.1281	0.4338	0.4752
TMT B	-0.2371	0.3590	-0.1082	-0.1179	0.4078	0.0247
BNT spontaneous recall	-0.1887	0.2703	-0.5576	-0.1300	-0.4681	-0.1346
**NCI group (n = 392)**						
	**PC1**	**PC2**	**PC3**	**PC4**	**PC5**	**PC6**
Standard deviation	2.3962	1.4179	1.1249	0.9771	0.9502	0.8920
Proportion of variance	0.3828	0.1340	0.0844	0.0637	0.0602	0.0530
Cumulative proportion of variance	0.3828	0.5168	0.6012	0.6648	0.7250	0.7781
Individual test loadings						
MMSE	-0.1033	0.2569	-0.0807	0.2215	0.8312	-0.3164
Clock test	-0.0857	0.4187	-0.4669	-0.2221	-0.0037	0.2391
Copy test	-0.0427	0.3620	-0.6225	0.0317	-0.1470	0.0698
AVLT 1	-0.2725	-0.1185	-0.0793	0.3971	-0.1515	0.1061
AVLT 2	-0.3411	-0.1028	-0.0246	0.2235	-0.0387	0.1217
AVLT 3	-0.3689	-0.1083	0.0042	-0.0018	-0.0122	0.0360
AVLT 4	-0.3658	-0.1307	-0.0038	-0.0182	0.0403	0.0145
AVLT 5	-0.3515	-0.0721	-0.0563	-0.1104	0.0426	0.0731
AVLT total	-0.4055	-0.1252	-0.0336	0.0942	-0.0194	0.0791
AVLT delayed	-0.3231	-0.1202	-0.0599	-0.2049	0.0512	-0.0404
AVLT recognition	-0.1926	-0.0750	0.0149	-0.7437	0.0230	-0.3323
Semantic fluency Animals	-0.1910	0.2635	0.1019	0.2485	-0.1818	-0.5921
TMT A	-0.1501	0.3627	0.4126	-0.1139	0.1677	0.3251
TMT B	-0.1276	0.4136	0.3973	-0.0374	-0.0088	0.3469
BNT spontaneous recall	-0.1220	0.4015	0.1854	0.0170	-0.4408	-0.3316

The six first PCs are presented from each PCA with values rounded to four decimals.

Supplementary Table 2. Principal component analysis by subgroups after propensity score

matching, MemClin.

CI group (n = 143)						
	**PC1**	**PC2**	**PC3**	**PC4**	**PC5**	**PC6**
Standard deviation	2.5793	1.6616	1.3282	1.1957	1.0906	1.0205
Proportion of variance	0.3326	0.1380	0.0882	0.0715	0.0595	0.0521
Cumulative proportion of variance	0.3326	0.4707	0.5589	0.6304	0.6898	0.7419
Individual test loadings						
MMSE	-0.1988	-0.1080	0.0587	-0.1530	0.2559	-0.0030
WAIS-IV Information	-0.1298	-0.0395	0.1048	-0.1516	0.1234	0.7551
WAIS-IV Block design	-0.1742	-0.2480	-0.2584	-0.1019	0.3309	0.0431
WAIS-IV Digit Span total	-0.1450	-0.3873	0.3153	0.2209	-0.0588	-0.1371
WAIS-IV Digit Span forward	-0.0792	-0.3317	0.3611	0.2444	-0.1733	0.0587
WAIS-IV Digit Span backward	-0.1210	-0.2652	0.3618	0.2657	0.1127	-0.1494
AVLT 1	-0.2829	0.1337	0.0979	0.0761	0.1170	-0.1240
AVLT 2	-0.3070	0.1999	0.1075	0.1975	0.1104	-0.1092
AVLT 3	-0.2974	0.2651	0.0445	0.1520	-0.0157	0.0118
AVLT 4	-0.3033	0.2720	0.0372	0.0623	-0.0658	-0.0244
AVLT 5	-0.2765	0.2981	-0.0128	0.0203	0.0028	0.1165
AVLT total	-0.3386	0.2674	0.0583	0.0998	0.0174	0.0088
RCFT copy	-0.0668	-0.1565	-0.0032	-0.1243	0.7314	-0.1314
D-KEFS verbal fluency FAS	-0.2083	-0.2186	0.0862	-0.1674	-0.1854	0.3260
D-KEFS semantic fluency	-0.2559	-0.1693	0.0231	-0.2147	-0.1335	0.1894
D-KEFS semantic fluency shifting (n correct answers)	-0.2587	-0.1101	-0.0233	-0.4408	-0.1806	-0.3041
D-KEFS sematic fluency shifting (n correct shiftings)	-0.2459	-0.1066	-0.0341	-0.4630	-0.2262	-0.2907
D-KEFS TMT 1	-0.1558	-0.1980	-0.3632	0.2493	-0.2421	-0.0078
D-KEFS TMT 2	-0.1673	-0.1599	-0.4736	0.2316	0.0396	-0.0196
D-KEFS TMT 3	-0.1806	-0.2081	-0.4052	0.2607	-0.0422	0.0703
**NCI group (n = 143)**						
	**PC1**	**PC2**	**PC3**	**PC4**	**PC5**	**PC6**
Standard deviation	2.4816	1.7304	1.3896	1.2732	1.1431	0.9989
Proportion of variance	0.3079	0.1497	0.0966	0.0811	0.0653	0.0499
Cumulative proportion of variance	0.3079	0.4576	0.5542	0.6352	0.7006	0.7505
Individual test loadings						
MMSE	-0.1808	0.0205	0.0990	0.0844	-0.2895	0.3132
WAIS-IV Information total	-0.1336	-0.2211	-0.2660	0.3220	-0.3035	0.0016
WAIS-IV Block Design total	-0.1521	-0.3192	-0.0710	0.1119	-0.3895	0.0099
WAIS-IV Digit Span total	-0.2008	-0.2993	0.3490	0.2186	0.1891	-0.0636
WAIS-IV Digit Span forward	-0.1590	-0.2314	0.4195	0.1737	0.1976	0.0732
WAIS-IV Digit Span backward	-0.1354	-0.1883	0.4554	0.1949	0.0663	-0.2893
AVLT 1	-0.2679	0.1209	0.0926	0.0427	0.1153	0.0244
AVLT 2	-0.2987	0.2470	0.0378	0.0316	-0.0670	0.0269
AVLT 3	-0.2727	0.3191	0.0107	0.0232	-0.1281	-0.0905
AVLT 4	-0.3020	0.2807	0.0031	0.0001	0.0411	-0.0802
AVLT 5	-0.2927	0.2702	-0.0095	0.0198	-0.0523	-0.0932
AVLT total	-0.3404	0.2976	0.0251	0.0267	-0.0233	-0.0582
RCFT copy	-0.1355	-0.1915	-0.2582	0.3432	-0.3464	-0.1807
D-KEFS verbal fluency FAS	-0.1924	-0.0926	-0.0806	0.1030	0.2350	0.6857

D-KEFS semantic fluency	-0.2502	-0.1383	-0.2887	0.0221	0.2414	0.2829
D-KEFS semantic fluency shifting (n correct answers)	-0.2437	-0.1726	-0.2865	-0.1547	0.3440	-0.2959
D-KEFS semantic fluency shifting (n correct shiftings)	-0.2310	-0.2233	-0.3211	-0.1214	0.2617	-0.2950
TMT 1	-0.1371	-0.1993	-0.0566	-0.3436	-0.0115	0.1187
TMT 2	-0.1711	-0.2340	0.1461	-0.4784	-0.2258	-0.0418
TMT 3	-0.1883	-0.1177	0.1792	-0.4917	-0.2875	0.0866

The six first PCs are presented from each PCA.
